# Flora of Forest Reserves and Riparian Areas in Benin

**DOI:** 10.3897/BDJ.13.e129992

**Published:** 2025-01-27

**Authors:** Eben-Ezer Apelete, Jean Cossi Ganglo

**Affiliations:** 1 Faculty of Agronomic Sciences (FSA), University of Abomey-Calavi (UAC), Abomey-Calavi, Benin Faculty of Agronomic Sciences (FSA), University of Abomey-Calavi (UAC) Abomey-Calavi Benin

**Keywords:** sacred forests, occurrence data, CERF, GBIF, PIFSAP

## Abstract

**Background:**

Benin government is committed to the overall strategy for the sacred forests and protected areas conservation through their integration into the national system of protected areas. This is due to the value of biodiversity and the considerable ethno-cultural and religious importance of the forests and protected areas. The project entitled “Project for the Integration of Sacred Forests into the System of Protected Areas of Benin (PIFSAP)” was initiated to preserve the biodiversity of the country by protecting and sustainably managing forest resources, biological and cultural heritage of local populations. Thus, all the sacred forests of Benin have been inventoried.

**New information:**

The dataset published on GBIF website contains 8,488 occurrences of species collected in the forests and protected areas of Benin. After cleaning, the dataset was made accessible to the whole world via GBIF website: https://www.gbif.org/dataset/0740c09f-5cf6-48f0-8e94-abaa46f4b87b. This dataset is one of the largest databases published on “Centre d’Etudes et de Recherche sur les Forêts (CERF)” account and can be used for any purpose. The data collected are unique and provides information on species diversity found in the forest reserves of Benin.

## Introduction

Benin is home to a large number of sacred forests which are subject to conservation and distinguished from each other by specific functions: convents of deities, shelters for spirits, gardens of medicinal plants or cemeteries. Benin has about 2940 sacred forests covering an area of 18360 ha (Kokou and Sokpon 2006, FAO 2020). These forest ecosystems are reservoirs of terrestrial biodiversity, both plant and animal (FAO 2020).

In Benin, forest biodiversity is important for households and exploited by populations in various fields: food, traditional medicine, energy and grazing (Koudokpon et al. 2018). Despite the lack of detailed information on the biological resources of the sacred forests, they contain many medicinal plants and endangered animal and plant species.

However, Benin, like other countries around the world, is experiencing a loss of biodiversity (RNCBDB 2014). On average, Benin loses fifty thousand hectares of forest per year (FAO 2020). This is mainly due to the extension of agriculture, overgrazing and the anarchic exploitation of resources (Hadonou-Yovo et al. 2019). Thus, the protection of sacred forests has proven to be an important tool for the sustainable conservation of biodiversity (RNCBDB 2014, FAO 2020).

Given the importance of the sacred forests for local populations, it is important to define conservation strategies and sustainable use of these resources. This is precisely what the authorities in charge of biodiversity conservation in Benin understood by initiating the project named Project for the Integration of Sacred Forests into the System of Protected Areas of Benin (PIFSAP) carried out from 2011 to 2016, aiming at conserving the biodiversity present in Benin. Through this project co-financed by the Global Environment Facility (GEF), the beneficiary municipalities, the United Nations Development Program (UNDP) and the Government of Benin, inventories have been carried out in almost all the sacred forests of Benin. Several sacred forests have now become conservatories of biodiversity. This dataset published on the GBIF website constitutes part of the data collected during the project.

## General description

### Purpose

Project for the Integration of Sacred Forests into the System of Protected Areas of Benin (PIFSAP) aims at improving the sustainable use of globally significant areas in and around the country by integrating them into the formal Protected Area (PA) system, strengthening legal and institutional protection and promoting community co-management of sacred forests. The project worked to reforest the degraded sacred forests in Benin. Thus, inventories have been done and the directory of sacred forests of Benin updated. This dataset collected and published later in GBIF website is very useful in guiding decision-making for the conservation and sustainable use of biodiversity in Benin.

## Project description

### Study area description

The data were collected in Benin, more precisely in the forests and reserves of the country.

The data collected during this study covers all the departments of Benin. More specifically, the country's forests and reserves were covered. Benin is located between 6º30' and 12º30' N and 1º and 3º40' E (Neuenschwander and Toko 2011). It is bordered to the North by Niger, to the East by Nigeria, to the West by Togo and to the North-West by Burkina-Faso. Benin covers a total area of 114763 km² with a 125 km long coastline and a straight-line distance of 700 km from the Atlantic to the Niger River in the North (Neuenschwander and Toko 2011).

In the south, monthly temperature averages vary between 26 °C and 28 °C (Neuenschwander and Toko 2011) while in the North, monthly maximum averages are above 35°C and can even reach 40 °C in Kandi (Neuenschwander and Toko 2011). Rainfall is between 900 mm and 1400 mm per year. From South to North, the vegetation presents specific characteristics to the following climatic formations: shrubby bush, wooded savannah, open forest and shrubby savannah (Azontondé 1991).

Benin has a forest cover composed of trees and shrubs representing 65% of its territory (Azontondé 1991). This vegetation is in constant decline. There are a few forest islands, wooded or shrubby savannahs, mangroves and aquatic meadows (Azontondé 1991).

### Design description

The study flora of forest reserves and riparian areas in Benin was performed with an integrated approach at both community and species levels. The standard methods of plant systematics and phytogeography were used.

### Funding

The Project for the Integration of Sacred Forests into the System of Protected Areas of Benin (PIFSAP) was co-financed by the Global Environment Facility (GEF), the United Nations Development Program (UNDP), the Government of Benin and the beneficiary municipalities.

## Sampling methods

### Study extent

The main stages of this project are documentary research, recruitment and training of field agents, fieldwork, data processing and cartographic drafting. The documentary review was carried out with the projects and technical services of the DGFRN, IGN and CERF. It made it possible to obtain the directory of sacred forests drawn up by Agbo and Sokpon in 1998, the occupation map of the study area and the base maps of the communes. The field equipment used during the inventories consists of: GPS receivers (Global Positioning System) for georeferencing (longitude and latitude), compass to take the direction of walking and orientate oneself and digital cameras for taking photos. The coordinate system used was WGS 84/UTM zone 31N.

### Sampling description

**Figure 1.**
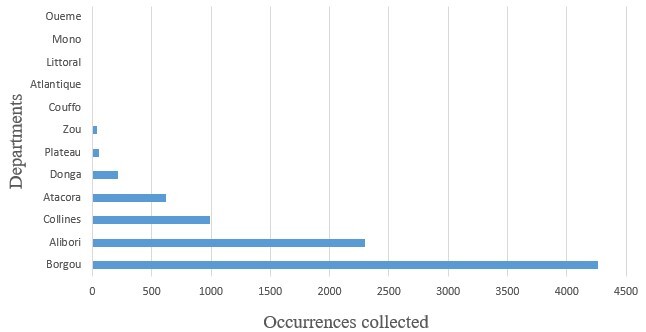
Occurrences collected by department.

An inventory of species in the sacred forests and reserves of Benin was carried out. Plant species occurrences were collected by random sampling in plots of one (1) ha (100 m x 100 m). The collection method is observation and only presence data were collected. All information collected was formatted in the GBIF spreadsheet. Some of the important information included: scientific name; date of data collection in terms of day, month and year; geographic coordinates in decimal degrees (longitude and latitude) and other information deemed important. In total, 8488 occurrences were collected in the municipalities visited (Borgou, Alibori, Collines, Atacora, Donga, Plateau, Zou, Couffo, Atlantique, Littoral, Mono and, Oueme) Fig. 1.

### Quality control

Herbariums of the species collected in the field were sent to the National Herbarium of Benin (HNB) for identification and validation of scientific names. The information noted on each species was entered into the spreadsheet (Excel sheet containing the standard fields for publishing data on the GBIF website) in accordance with those set by the Darwin Core (DwC) Standard (Darwin Core Task Group 2009). Finally, the dataset was cleaned and prepared for publication on the GBIF website.

The data collected were, therefore, cleaned according to the following steps: define and determine the types of errors; search for and identify occurrences of errors; correct the errors; documenting error cases and typical errors and modifying the data entry process to reduce future errors.

### Step description

The information collected from the inventory of species in the sacred forests and reserves of Benin made it possible to properly complete the spreadsheet (Wieczorek et al. 2012). Plant species occurrences were collected by random sampling in plots of one (1) ha (100 m x 100 m). The collection method is observation and only presence data were collected.

Taxonomic, temporal and spatial data (Ganglo et al. 2017) have been corrected. For taxonomic errors, Catalogue of life and Taxonomic Name Resolution Service (TRNS) was used to correct spelling errors (badly written names), format errors (binomial nomenclature) and to replace the names given in synonymy with those accepted. Regarding errors related to spatial data, geographical coordinates (longitude and latitude) were projected on QGIS Desktop software version 2.18.4 (Sutton and Dassau 2015) and those outside of the study area (outlier) were deleted (Ganglo et al. 2017). Occurrences with no collection location information were removed. *Geolocate* and *Eathexplorer* were used to find geographical coordinates from administrative subdivisions or from the description of the place of collection. In terms of temporal errors, error in dates such as 13-13-2050 (errors made during entry) have been corrected from the field *evenDate*. Feedback was, therefore, given to the data provider to this effect. Moreover, the field *evenDate* have been formatted under the standard accepted for publication on GBIF website: *Year, month and day*. Finally, the duplicates occurrences have been removed by considering the fields *scientifcName, decimalLatitude* and *decimalLongitude* (fields relating to essential attributes of primary biodiversity data in order to have unique occurrence).

## Geographic coverage

### Coordinates

9.974 and 10.014 Latitude; 3.651 and 1.397 Longitude.

## Taxonomic coverage

### Description

**Figure 2.**
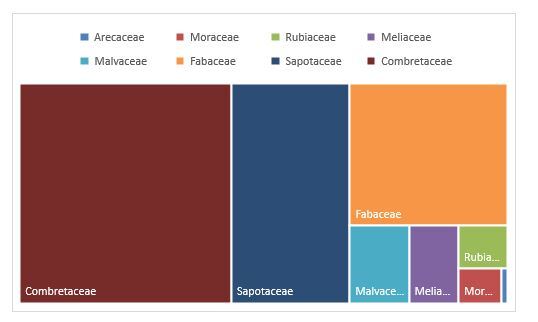
Record by family.

All the species collected were identified at the National Herbarium of Benin (NHB) and all belong to the Tracheophyta phylum and the Magnoliopsida class. The dataset has a total of eight (8) families: Combretaceae (3689 occurrences), Sapotaceae (2060 occurrences), Fabaceae (1775 occurrences), Malvaceae (362 occurrences), Meliaceae (303 occurrences), Moraceae (118 occurrences), Rubiaceae (166 occurrences) and Arecaceae (15 occurrences). The Combretaceae family, therefore, has the most occurrences collected for this dataset, followed by the Sapotaceae family (Fig. 2). In total, nineteen (19) genera have been listed from the dataset. The main genera with the largest number of occurrences collected are respectively *Vitellaria* (2060 occurrences), *Terminalia* (2044 occurrences) and *Anogeissus* (1645 occurrences). Knowing *Vitellaria* as the most represented genus in the dataset, the graph below informs us that the species *Vitellariaparadoxa* is the most abundant (24.27 % of the dataset).

## Temporal coverage

**Data range:** 2013-1-01 – 2013-12-29.

### Notes

**Figure 3.**
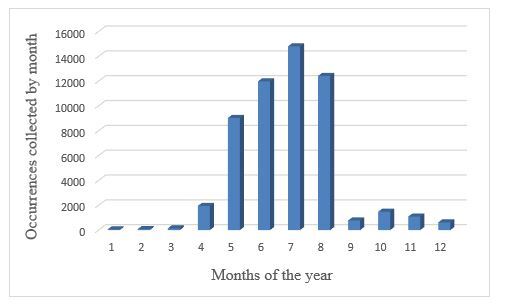
Histogram by month.

This dataset has been collected during all the months of 2013. The number of occurrences collected varies by month. We still observe a peak in the month of July followed by the month of August (Fig. 3).

## Usage licence

### Usage licence

Open Data Commons Attribution License

### IP rights notes

The license used for this dataset is Attribution 4.0 International (CC-BY 4.0). This license means that you are free to share (copy and redistribute the material in any medium or format) and adapt (remix, transform, and build upon the material for any purpose, even commercially) the data.

Under the following terms:

- **Attribution**: you must give appropriate credit, provide a link to the license and indicate if changes were made. You may do so in any reasonable manner, but not in any way that suggests the licensor endorses you or your use.
- **No addition restrictions**: You may not apply legal terms or technological measures that legally restrict others from doing anything the license permits.

Important things you have to notice is that you do not have to comply with the licene for elements of the material in the public domain or where your use is permitted by an applicable exception or limitation. Moreover, no warranties are given. The license may not give you all of the permissions necessary for your intended use. For example, other rights such as publicity, privacy or moral rights may limit how you use the material

## Data resources

### Data package title

Study of specific plant diversity in forest reserves and riparian areas in Benin

### Resource link

https://doi.org/10.15468/dl.9xgpx8

### Alternative identifiers

https://www.gbif.org/dataset/0740c09f-5cf6-48f0-8e94-abaa46f4b87b

### Number of data sets

1

### Data set 1.

#### Data set name

Study of specific plant diversity in forest reserves and riparian areas in Benin

#### Data format

Data format is DarwinCore

#### Download URL

https://ipt.gbifbenin.org/archive.do?r=flordata

#### Description

The dataset contains 8488 occurrences of plant species from Benin, representing the following families: Combretaceae, Sapotaceae, Fabaceae, Malvaceae, Meliaceae, Moraceae, Rubiaceae and Arecaceae (https://doi.org/10.15468/dl.9xgpx8)

**Data set 1.** 

Column label	Column description
occurrenceID	An unique identifier for the occurrence.
occurrenceID	An identifier for the Occurrence (as opposed to a particular digital record of the occurrence). In the absence of a persistent global unique identifier, construct one from a combination of identifiers in the record that will most closely make the occurrenceID globally unique.
institutionCode	The name (or acronym) in use by the institution having custody of the object(s) or information referred to in the record.
basisOfRecord	The specific nature of the data record.
eventDate	The date-time or interval during which an Event occurred. For occurrences, this is the date-time when the event was recorded. Not suitable for a time in a geological context.
year	The four-digit year in which the Event occurred, according to the Common Era Calendar.
month	The integer month in which the Event occurred.
day	The integer day of the month on which the Event occurred.
kingdom	The full scientific name of the kingdom in which the taxon is classified.
phylum	The full scientific name of the phylum or division in which the taxon is classified.
class	The full scientific name of the class in which the taxon is classified.
order	The full scientific name of the order in which the taxon is classified.
family	The full scientific name of the family in which the taxon is classified.
genus	The full scientific name of the family in which the taxon is classified.
specificEpithet	The full scientific name of the family in which the taxon is classified.
scientificName	The full scientific name, with authorship and date information if known.
scientificNameAuthorship	The authorship information for the scientificName formatted according to the conventions of the applicable nomenclaturalCode.
taxonRank	The taxonomic rank of the most specific name in the scientificName.
continent	The name of the continent in which the Location occurs.
countryCode	The standard code for the country in which the Location occurs.
country	The name of the country or major administrative unit in which the Location occurs.
stateProvince	The name of the next smaller administrative region than country (state, province, canton, department, region etc.) in which the Location occurs.
locality	The specific description of the place.
decimalLatitude	The geographic latitude (in decimal degrees, using the spatial reference system given in geodeticDatum) of the geographic center of a Location. Positive values are north of the Equator, negative values are south of it. Legal values lie between -90 and 90, inclusive.
decimalLongitude	The geographic longitude (in decimal degrees, using the spatial reference system given in geodeticDatum) of the geographic center of a Location.
geodeticDatum	The ellipsoid, geodetic datum or spatial reference system (SRS), upon which the geographic coordinates given in decimalLatitude and decimalLongitude ares based.
habitat	A category or description of the habitat in which the Event occurred.
occurrenceStatus	A statement about the presence or absence of a Taxon at a Location.
samplingProtocol	The methods or protocols used during an Event, denoted by an IRI.
recordedBy	A person, group or organization responsible for recording the original Occurrence.
parentEventID	An identifier for the broader Event that groups this and potentially other Events.
ownerInstitutionCode	The name (or acronym) in use by the institution having ownership of the object(s) or information referred to in the record.
